# The Effect of Outdoor Activity Intention on Depressive Mood: The Mediating Role of Outdoor Activity Frequency

**DOI:** 10.3390/healthcare13233047

**Published:** 2025-11-25

**Authors:** Fuxiang Yu, Chuntian Lu, Zhengbing Guo

**Affiliations:** 1 Business School, Hangzhou City University, Hangzhou 310015, China; yufx@hzcu.edu.cn; 2 School of Humanities and Social Science, Xi’an Jiaotong University, Xi’an 710049, China; luchuntian@xjtu.edu.cn

**Keywords:** outdoor activity intention, outdoor activity frequency, depressive mood, theory of planned behavior, mental health

## Abstract

**Background:** With rapid urbanization, mental health challenges such as depression and emotional distress are becoming increasingly common. Contact with natural environments has been shown to improve mental health, yet most studies focus on direct associations between environmental exposure and mental health. The behavioral mechanisms underlying these benefits remain underexplored. This study aims to investigate whether outdoor activity frequency mediates the relationship between individuals’ intention to engage in outdoor activities and depressive mood. **Methods:** We used data from the 2021 Chinese General Social Survey (CGSS). Drawing on the Theory of Planned Behavior (TPB) and supported by Stress Recovery Theory (SRT) and Attention Restoration Theory (ART), we constructed a mediation model. Ordinary Least Squares regression, ordered logistic regression, and mediation analysis were applied to assess the relationships among outdoor activity intention, activity frequency, and depressive mood. **Results:** The analyses revealed three main findings. First, outdoor activity intention alone did not directly reduce depressive mood. Second, outdoor activity frequency significantly alleviated depressive mood, suggesting that actual behavioral engagement with nature is critical. Third, the effect of outdoor activity intention on depressive mood was fully mediated by activity frequency. **Conclusions:** The results demonstrate that the mental health benefits of natural environments are not automatically derived from intention or preference but require active and frequent engagement. These findings provide empirical support for behavior-based interventions in urban mental health strategies and establish a theoretical foundation for future research on the pathways linking nature and mental health.

## 1. Introduction

Mental health disorders have become a critical global public health issue, with depression, anxiety, and emotional disorders accounting for an increasing share of the global disease burden [1,2]. In modern urban environments, the fast pace of life, high population density, environmental stressors, and social isolation have driven the growing prevalence of mental health challenges [3,4]. Against this backdrop, the role of nature contact in promoting mental health has attracted substantial academic and policy interest [5]. A growing body of research consistently shows that exposure to natural environments offers significant mental health benefits [6,7,8]. These benefits include stress reduction and emotional restoration [9,10,11]. Nevertheless, existing studies have predominantly focused on the outcome-oriented relationship between objective environmental attributes (e.g., green space coverage, blue space quality) and mental health [12], often neglecting the individual behavioral pathways through which nature exerts its mental health benefits [13,14]. Fundamentally, the relationship between nature and mental health is not a passive exposure–response dynamic but rather an active behavioral process [15]. Individuals interact with natural environments through purposeful activities such as walking, hiking, or recreational visits, making behavior the core mechanism through which the mental health benefits of nature are realized [16,17].

A substantial body of research has confirmed that engaging in outdoor activities is associated with reduced depressive symptoms and improved well-being [18]. Empirical studies have shown that activities such as walking, cycling, or gardening can effectively alleviate stress, anxiety, and fatigue by promoting physical movement and social interaction [19,20]. However, most of these studies conceptualize outdoor activity primarily as a form of physical exercise, emphasizing its physiological effects such as increased endorphin production or cardiovascular activation. In contrast, the present study approaches outdoor activity from the perspective of environmental psychology, viewing it as a deeper act of embracing nature rather than a mode of physical training. Our research therefore dialogues with studies on urban green space and mental health, focusing on how meaningful contact with nature through sensory, emotional, and cognitive engagement supports psychological restoration. In this framework, outdoor activity is understood as an embodied expression of connecting with nature, serving as a bridge between human intention and the restorative qualities of natural environments. Nevertheless, a more nuanced understanding of how subjective intentions translate into actual engagement with nature remains limited. Individuals may hold positive attitudes toward outdoor activities but fail to act on these intentions because of personal, social, or environmental barriers [21]. As a result, converting outdoor activity intentions into tangible behaviors becomes a critical step in harnessing nature’s mental health benefits [22,23]. Despite its significance, this intention–behavior–health linkage has received limited empirical attention, particularly in non-Western contexts, and therefore warrants further exploration. In line with the core aim of this study, which is to examine whether embracing nature can yield measurable mental health benefits, we treat outdoor activity intention and actual outdoor interaction as two key dimensions of this embracing process.

The Theory of Planned Behavior (TPB) offers a robust theoretical framework to understand the gap between intention and behavior. TPB posits that behavioral intention is the most immediate antecedent of actual behavior [24,25]. Within the context of outdoor activities, TPB suggests that fostering strong intentions is necessary but not sufficient, and actual engagement depends on the perceived ease or difficulty of performing the behavior [26]. In this study, TPB serves as the conceptual lens through which outdoor activity intention is examined as a precursor to behavior, providing a theoretical basis for analyzing the translation of cognitive readiness into frequent outdoor activities. While the TPB offers a valuable lens for understanding how individual attitudes and intentions shape outdoor behavior, the psychological benefits derived from such behavior are more comprehensively explained through environmental psychology frameworks—specifically, the Stress Recovery Theory (SRT) and the Attention Restoration Theory (ART). These two theories jointly elucidate the multidimensional restorative effects of nature on human well-being. According to SRT, natural environments evoke an automatic, affective, and physiological recovery from stress by reducing negative arousal and promoting positive emotional states [27]. Empirical research has demonstrated that exposure to natural scenes can lower blood pressure, decrease cortisol levels, and induce feelings of calmness and safety, which together contribute to reduced depressive mood and emotional stabilization [28]. In this sense, outdoor activities represent not only behavioral choices but also embodied pathways through which individuals experience these psychophysiological benefits of stress reduction in real-life contexts. In contrast, ART focuses on the cognitive dimension of restoration. It posits that contact with nature replenishes depleted attentional resources by providing softly fascinating stimuli that engage effortless attention, allowing directed attention mechanisms to rest and recover [29]. Such cognitive restoration alleviates fatigue, enhances concentration, and contributes to improved emotional regulation and overall mental health [30]. Therefore, outdoor activity is not merely a leisure pursuit but a behavioral expression of attentional renewal and psychological rebalancing.

Integrating these perspectives, this study proposes that the frequency of outdoor activity functions as a key behavioral mediator connecting intention with mental health outcomes. While TPB explains why individuals form intentions to engage with outdoor environments, SRT and ART clarify how repeated and embodied interactions with nature—manifested through frequent outdoor activities—translate those intentions into tangible psychological benefits. By situating the behavioral mechanism of outdoor engagement within these dual restorative frameworks, the study bridges motivational theory with environmental psychology to advance a more comprehensive understanding of how nature-based behaviors alleviate depressive mood (DM).

To summarize, this study aims to explore the behavioral mechanisms connecting outdoor activity intention to mental health, with a focus on the mediating role of activity frequency. Specifically, it addresses two key questions: first, whether outdoor activity intention significantly alleviates DM, and second, whether the outdoor activity frequency mediates the relationship between intention and DM. Accordingly, we formulate the following hypotheses:

**H1.**  *Outdoor activity intention is negatively associated with depressive mood.*

**H2.**  *Outdoor activity intention positively predicts outdoor activity frequency.*

**H3.**  *Outdoor activity frequency is negatively associated with depressive mood.*

**H4.**  *Outdoor activity frequency mediates the relationship between outdoor activity intention and depressive mood.*

## 2. Materials and Methods

### 2.1. Analytical Framework

To summarize the conceptual logic guiding this research, the study aims to explore the behavioral mechanisms connecting outdoor activity intention to DM, emphasizing the mediating role of outdoor activity frequency. Drawing upon TPB, activity intention is interpreted as a cognitive precursor to actual behavior, while SRT and ART provide complementary explanations for how nature-based engagement promotes psychological restoration. This cross-theoretical framework enables a more comprehensive understanding of the interplay between cognition, behavior, and DM. Specifically, TPB captures the motivational readiness underlying nature-oriented behavior, whereas SRT and ART explain how repeated nature interactions foster emotional recovery and cognitive restoration.

The present study makes three main contributions. First, it integrates behavioral psychology with environmental psychology, connecting cognitive intention, behavioral practice, and mental health outcomes. Second, it advances an embodied practice perspective, conceptualizing outdoor activity as an active form of “embracing nature” rather than passive exposure, thereby highlighting the agency of individuals in realizing nature’s restorative potential. Third, it offers practical implications for urban mental health promotion, suggesting that green space planning and behavioral interventions should prioritize facilitating consistent outdoor engagement as a pathway to mental well-being.

### 2.2. Data Collection

The data employed in this study were derived from the 2021 wave of the Chinese General Social Survey (CGSS), administered by the National Survey Research Center at Renmin University of China. The CGSS is a nationally representative, large-scale, and continuous social survey that adopts a multistage stratified probability sampling design, covering both urban and rural residents across 31 provinces, autonomous regions, and municipalities in mainland China (excluding Hong Kong, Macao, and Taiwan). The 2021 survey, which was the 14th annual wave, was conducted over nearly five months in collaboration with partner universities across the country. The project team overcame multiple challenges posed by the COVID-19 pandemic to complete the fieldwork. The 2021 dataset provides rich information on individuals’ mental health, outdoor activity engagement, behavioral intentions, and various sociodemographic characteristics. To ensure data quality, we excluded respondents with missing values on key variables such as outdoor activity intention, frequency of outdoor behavior, DM, and control variables. After data cleaning, a total of 2583 valid observations were retained for the empirical analysis.

### 2.3. Variables

#### 2.3.1. Dependent Variable

In this study, depressive mood is conceptually distinct from clinically diagnosed depression. Rather than capturing a medical condition that requires professional assessment and multiple diagnostic tests, our measure reflects subjective self-reports of depressive mood, which indicate the perceived frequency of negative emotional states. Specifically, DM is measured using a single item from the CGSS 2021 questionnaire: “In the past four weeks, how often have you felt depressed?” Responses range from 1 (“never”) to 5 (“always”). Strictly speaking, mental health includes both positive aspects, such as happiness and life satisfaction, and negative aspects, such as depression and anxiety [31,32]. In this study, we adopt a negative definition of mental health, focusing on the alleviation of psychological distress. This approach aligns with the Stress Reduction Theory and Attention Restoration Theory, both of which emphasize the benefits of nature exposure for reducing negative psychological states. SRT proposes that natural environments help diminish physiological stress responses, while ART suggests that natural settings can restore cognitive resources depleted by urban life and information overload. These theoretical foundations justify our use of DM as the primary mental health indicator in this analysis. This approach is consistent with other studies that have used single-item self-reported measures of mental health based on national survey data [33]. By clearly distinguishing depressive mood from clinically diagnosed depression, we ensure that the scope of this study aligns with the available data and avoids overstating the medical implications of our findings.

#### 2.3.2. Independent Variable

The independent variable in this study is self-reported outdoor activity intention, which reflects individuals’ motivational readiness to engage with natural environments. Drawing on the Theory of Planned Behavior, this variable captures the attitudinal component that precedes behavioral action. Respondents were asked, “If possible, how much would you enjoy engaging in outdoor activities in nature?” Responses were recorded on a five-point Likert scale ranging from 1 = “Not at all” to 5 = “Very much.” A higher score denotes a stronger personal preference and greater intention to participate in nature-based outdoor activities. It is important to note that this measure indicates perceived intention rather than objectively observed or clinically assessed motivation.

#### 2.3.3. Mediating Variable

The mediating variable is self-reported outdoor activity frequency, which reflects the behavioral enactment of the intention to engage with nature in Table 1. Respondents were asked to report how often they participated in outdoor leisure activities such as hiking, bird-watching, swimming, or skiing during the past 12 months. This was measured on a five-point scale where 1 = “Never”, 2 = “Rarely”, 3 = “Sometimes”, 4 = “Often”, and 5 = “Almost every day.” A higher score indicates more frequent contact with natural environments. We acknowledge that this variable captures subjective reports of activity frequency rather than objectively recorded behavior.

#### 2.3.4. Control Variables

All models controlled for demographic variables and social determinants of DM. The demographic variables included age, ethnicity, gender, marital status, physical condition, political status, and interview site [34,35,36,37]. Social determinants of DM encompassed family economic status and education level [38,39]. These variables were selected for three main reasons. First, prior research consistently indicates that demographic characteristics are systematically associated with mental health outcomes. For instance, women and older adults tend to report higher depressive symptoms [40], while married individuals often experience greater emotional support that buffers against psychological distress [41]. Second, physical condition and socioeconomic factors, including family economic status and education level, represent critical determinants of emotional well-being. Poorer health status can exacerbate physiological stress responses and limit participation in outdoor activities, whereas higher education and better economic standing often enhance coping skills and access to supportive environments [42]. Third, in the Chinese context, ethnicity, political status, and interview site capture key structural and institutional disparities that may shape psychological health. China’s multi-ethnic composition entails cultural and community-level variations in social support, lifestyle, and environmental exposure that may influence depressive symptoms. Meanwhile, political affiliation often reflects differences in institutional participation and access to collective resources, potentially affecting mental well-being. The interview site variable, which distinguishes between urban neighborhood committees and rural village committees, further accounts for spatial heterogeneity in public service provision [43].

Age is treated as a continuous variable, ranging from 18 to 94 years, with a mean of 51.2 and a standard deviation of 17.6. Gender is categorized as male (coded as 1) or female (coded as 0). Marital status is classified as married (coded as 1) or other (coded as 0). Political status includes members of the Communist Party, Communist Youth League, or Democratic Party (coded as 1) and the masses (coded as 0). Ethnicity is classified into ethnic minorities (coded as 1) and Han (coded as 0). Physical condition is a continuous variable rated from 1 to 5, with higher scores indicating better health. Family economic status reflects local income levels, measured as a continuous variable from 1 to 5; higher scores represent economic status above the local average. Household registration type differentiates between agricultural families (coded as 1) and other types (coded as 0). Education level is categorized from 1 to 4, where primary school and below is coded as 1, junior high school or equivalent as 2, high school or equivalent as 3, and undergraduate and above as 4. The interview sites were categorized into neighborhood committees (coded as 0) and village committees (coded as 1).

### 2.4. Statistical Analysis

A descriptive analysis was initially performed on the dependent, independent, mediating, and control variables to provide a general overview of the sample characteristics. Next, Ordinary Least Squares (OLS) regression was used to examine the direct relationship between outdoor activity intention and DM. Models 1–4 present the OLS regression results, focusing on the effects of outdoor activity intention and outdoor activity frequency on DM. To further validate the robustness of these findings, we conducted ordered logistic regression (Models 5–7), which confirmed the consistency of the relationships identified in the OLS models. Subsequently, we examined the mediating role of outdoor activity frequency in the relationship between outdoor activity intention and DM using PROCESS Model 4. Additionally, to assess the robustness of the findings, we conducted a sensitivity analysis using a randomly selected 50% subsample. The results aligned with those of the full sample, reinforcing the reliability of our conclusions. All analyses were conducted using SPSS version 25, and two-tailed *p*-values ≤ 0.05 were considered statistically significant.

## 3. Results

### 3.1. Descriptive Analysis

Table 2 summarizes the basic characteristics of the sample (N = 2583). The gender distribution was relatively balanced, with a slight female majority (54.2%). Most respondents were married (68.9%) and of Han ethnicity (92.5%). In terms of socioeconomic background, nearly half of the participants rated their family economic status as average (49.9%), and the majority had education levels of junior high school or below. Regarding health status, over half of the respondents perceived themselves as somewhat or very healthy. The sample included both urban and rural residents, with 56.2% surveyed in neighborhood committees and 43.8% in village committees.

### 3.2. OLS and Ordered Logistic Regression

Table 3 presents the results of the OLS regression models testing the hypothesized relationships among outdoor activity intention, outdoor activity frequency, and DM.

In Model 1, which included only the control variables, several demographic and socioeconomic characteristics showed significant associations with DM. Gender had a notable negative effect (β = −0.190, *p* < 0.001), indicating that females reported higher DM than males. Age was also negatively related to DM. Regarding socioeconomic factors, family economic status (β = −0.151, *p* < 0.001) and physical condition (β = −0.400, *p* < 0.001) both exerted strong influences on depressive mood. The interview site variable was also significantly associated with DM (β = −0.164, *p* < 0.001), indicating that respondents from rural village committees reported lower DM compared with those from urban neighborhood committees.

In Model 2, after including outdoor activity intention as an independent variable, the coefficient is negative but not statistically significant (β = −0.004, SE = 0.016), suggesting no direct effect of intention on DM. Therefore, H1 is not supported. In Model 3, where outdoor activity frequency serves as the dependent variable, outdoor activity intention shows a strong and significant positive effect (β = 0.271, SE = 0.020, *p* < 0.001). This indicates that individuals with stronger intentions to engage in outdoor activities tend to report higher actual participation frequency. Thus, H2 is strongly supported. In Model 4, the coefficient of frequency is significantly negative (β = −0.036, SE = 0.016, *p* < 0.05), confirming that higher outdoor activity frequency relates to lower DM. H3 is supported. In the same model, intention becomes slightly positive and remains non-significant. This pattern may indicate a mediation effect, in which the mental health benefit of outdoor activity intention operates indirectly through actual behavioral engagement. Variance inflation factor checks for all explanatory variables indicated no evidence of multicollinearity (all VIF values were below 2.5), and the small change in coefficient sign is likely attributable to statistical noise. Across all models, the adjusted R^2^ remains consistent at approximately 0.22, suggesting that the predictors together explain a moderate portion of the variance in DM.

To further verify the robustness of the structural relationship, we employed ordered logit regression models, as shown in Table 4. The results of the ordered logit regression are largely consistent with the OLS findings. Model 5 includes only control variables. In Model 6, outdoor activity intention is included but shows no significant association with DM (β = −0.027, SE = 0.031, *p* > 0.1). In Model 7, both outdoor activity intention and frequency are entered simultaneously. The coefficient for intention remains non-significant (β = −0.009, SE = 0.032), while frequency remains significantly associated with lower DM (β = −0.068, SE = 0.031, *p* < 0.05). The pseudo R^2^ remains stable across models (0.206–0.207), indicating modest explanatory power. These results reinforce the idea that actual engagement in outdoor activities, rather than mere intention, plays a crucial role in reducing DM.

### 3.3. Mechanism Analysis

To further explore the mechanism linking outdoor activity intention to DM, we conducted a mediation analysis with outdoor activity frequency as the mediating variable. The results, presented in Table 5, reveal a significant indirect effect of outdoor activity intention on DM through activity frequency (β = −0.0096, SE = 0.0044, 95% CI [−0.0183, −0.0008]), as the confidence interval does not include zero. This indicates that individuals who express a greater intention to engage in outdoor activities tend to participate more frequently, which in turn reduces their DM. However, the direct effect of outdoor activity intention on DM is not statistically significant (β = 0.0053, SE = 0.0163, 95% CI [−0.0265, 0.0372]), nor is the total effect (β = −0.0043, SE = 0.0157, 95% CI [−0.0351, 0.0265]). The combination of a significant indirect effect with non-significant direct and total effects indicates full mediation in the full-sample analysis, meaning that the influence of outdoor activity intention on DM operates entirely through actual behavioral engagement. This underscores the importance of not only cultivating positive intentions but also facilitating the translation of these intentions into regular practice.

### 3.4. Robustness Check: 50% Sample Validation

To address concerns regarding the potential limitations of the full sample size (N = 2583) in representing the national population of China, a robustness check was conducted by randomly selecting 50% of the original sample (N = 1310) to re-estimate the ordered logistic regression and mediation models. The goal was to assess whether the observed relationships remained consistent and reliable under a reduced data condition.

As shown in Table 6, the results of the ordered logistic regression using the 50% subsample were largely consistent with those obtained from the full sample. Specifically, outdoor activity frequency remained significantly negatively associated with depressive symptoms, while outdoor activity intention was not statistically significant in either model. This finding reinforces the robustness of the mediating role of outdoor activity behavior. The mediation analysis based on the 50% sample further confirmed the indirect pathway through outdoor activity frequency. As shown in Table 7, the indirect effect of outdoor activity intention on DM via frequency remained statistically significant (β = −0.0171, SE = 0.0064, 95% CI [−0.0297, −0.0049]), while the direct effect and the total effect were non-significant. The combination of a significant indirect effect with non-significant direct and total effects indicates full mediation in the 50% subsample, meaning that the influence of outdoor activity intention on DM operates entirely through behavioral engagement.

## 4. Discussion

### 4.1. Relationship Between Nature Engagement and DM

The findings of this study provide robust empirical evidence that frequent engagement with nature effectively alleviates DM, reinforcing and extending the foundational assumptions of ART and SRT. According to ART, natural environments gently capture involuntary attention, facilitating the recovery of cognitive resources often depleted by urban demands. Similarly, SRT posits that exposure to natural settings evokes positive affective responses and reduces physiological stress. In our analysis, outdoor activity frequency was significantly associated with lower DM, confirming that the mental health benefits of nature arise from embodied behavioral contact rather than mere exposure or passive viewing. This finding is consistent with prior evidence that habitual interaction with green spaces enhances affect regulation, reduces stress, and improves mental resilience [44,45]. Furthermore, a recent meta-analysis has demonstrated that individuals who frequently engage with natural environments exhibit substantially lower levels of anxiety and depression [46].

While ART emphasizes cognitive recovery and SRT highlights affective restoration, our findings reveal that these mechanisms are not isolated but operate synergistically through behavior. Individuals who regularly participate in outdoor activities experience both emotional relaxation and cognitive rejuvenation, indicating that psychological restoration is not an automatic outcome of proximity to nature but a behavioral process requiring active engagement. The mediation effect observed in this study, where outdoor activity frequency serves as a bridge linking intention to depressive mood, further clarifies that the restorative potential of nature is behaviorally activated [47]. This finding refines the conceptual understanding of ART and SRT by demonstrating that restorative benefits emerge through sustained and intentional interaction with natural environments rather than passive spatial exposure.

From a behavioral health perspective, consistent engagement with nature is essential for sustaining psychological well-being. Neither positive intention nor one-time contact with nature is sufficient; mental recovery accumulates through repeated behavioral interaction. Frequent participation creates positive feedback loops in which improved mood motivates continued engagement, generating a self-reinforcing cycle of restoration. This interpretation echoes evidence from behavioral medicine showing that routine outdoor activity and recreational walking are strong predictors of reduced depression and anxiety [48,49].

Finally, the Chinese context adds further nuance to this mechanism. Rapid urbanization, constrained living environments, and long working hours restrict opportunities for regular outdoor activity, especially among urban residents. Meanwhile, structural disparities between urban and rural areas, rooted in the hukou system and unequal allocation of green infrastructure, further widen the gap in access to restorative environments. Residents in rural regions may enjoy proximity to nature but lack formal recreational facilities, whereas urban populations face spatial and temporal constraints despite higher service accessibility. These conditions reflect the broader structural inequalities that mediate the behavioral realization of nature’s mental health benefits. Therefore, policy measures that institutionalize equitable and sustained opportunities for outdoor engagement are essential for transforming sporadic exposure into continuous behavioral practices that support long-term mental health.

### 4.2. Intention as a Driver of Behavior: Expanding the Theory of Planned Behavior

The findings provide nuanced evidence that extends the theoretical scope of both TPB and environmental psychology frameworks. While TPB posits that behavioral intention predicts subsequent action, the current results reveal that intention alone does not significantly reduce depressive mood once actual behavioral frequency is considered [50]. This suggests that intention serves as a motivational prerequisite rather than a direct determinant of psychological outcomes [51]. In contrast, the mediating effect of outdoor activity frequency validates SRT and ART, which emphasize the restorative functions of repeated, embodied contact with nature. These results indicate that the mental health benefits of outdoor engagement emerge not merely from holding pro-environmental attitudes or intentions, but from the consistent behavioral enactment of nature-based experiences that activate both physiological and cognitive recovery pathways. In this way, the study bridges motivational and restorative theories, highlighting the necessity of behavioral implementation for realizing the psychological benefits proposed by SRT and ART.

### 4.3. Implications for Nature-Based Mental Health Promotion

The behavioral implications of this study are grounded in the empirical evidence that outdoor activity frequency, rather than intention alone, significantly reduces depressive mood. This finding suggests that interventions should not stop at motivating people to value outdoor experiences but should focus on facilitating behavioral execution—for example, by improving accessibility to nearby green and blue spaces, providing safe and convenient walking environments, and integrating outdoor activities into daily routines. Public health programs could also promote structured or community-based outdoor initiatives that help transform positive intentions into regular behaviors.

### 4.4. Limitations and Directions for Future Research

While this study contributes valuable insights into the role of outdoor activity intention and behavior in mental health, several limitations must be acknowledged. First, the cross-sectional design limits our ability to infer causal relationships between outdoor activity intention, outdoor activity frequency, and DM. Although the mediation model was tested using PROCESS and yielded robust results across multiple specifications, these analyses cannot determine temporal precedence. The observed associations should therefore be interpreted as correlational rather than causal. Future research should adopt longitudinal or experimental designs to verify the directionality and causal nature of the proposed pathways.

Second, this study relied on single-item self-reported measures for outdoor activity intention, outdoor activity frequency, and depressive mood, which inevitably limit construct validity. The use of single-item measures may restrict reliability and reduce the ability to capture multidimensional nuances of the underlying phenomena. While our operationalization follows the Theory of Planned Behavior by distinguishing between attitudinal intention and behavioral frequency, these measures reflect subjective perceptions rather than objectively observed or validated behavioral data. The measurement of outdoor activity frequency used in this study represents a simplified indicator that does not distinguish frequency, duration, or regularity of engagement. This operational conflation may obscure meaningful differences in total environmental exposure and its influence on physiological and psychological mechanisms, including circadian rhythm regulation. For example, two individuals reporting similar frequencies may differ substantially in how long or how regularly they spend time outdoors, potentially resulting in distinct mood-related outcomes. Future research should therefore employ objective and time-sensitive measures such as GPS tracking, accelerometers, or digital activity logs to differentiate between the frequency, duration, and regularity of outdoor engagement, allowing for a more nuanced assessment of nature’s impact on depressive mood.

Third, this study applied only the intention component of the Theory of Planned Behavior without incorporating measures of subjective norms or perceived behavioral control. This reflects our theoretical focus on linking intention to behavior as part of embracing nature, but such partial operationalization may limit the ability to fully capture all determinants of behavior as conceptualized in the Theory of Planned Behavior. Future research should incorporate the complete set of Theory of Planned Behavior components for a more comprehensive theoretical test.

Finally, although the sample of 2583 valid observations is relatively large for social survey research, it may still be insufficient to fully capture the complexity of participants’ psychological states. Our measure of DM is based on a single self-reported item, not a validated multi-item instrument. Therefore, it should not be interpreted as clinically diagnosed depression, but rather as subjective self-reports of depressive mood, reflecting perceived negative emotional states. Additionally, due to data constraints, this study could not account for seasonal variations in environmental conditions. Seasonal changes in daylight duration, temperature, and weather patterns are known to significantly influence both outdoor activity behavior and mood outcomes. Moreover, light exposure itself is a fundamental mechanism through which outdoor environments exert psychological effects, as it plays a key role in regulating circadian rhythms, serotonin synthesis, and other physiological processes associated with mood regulation. Because the CGSS 2021 survey was administered over several months and across diverse geographic regions, unobserved seasonal and photoperiodic heterogeneity may have affected respondents’ outdoor activity intentions, frequencies, and depressive symptoms. This limitation restricts the generalizability of the findings. Future research should therefore integrate temporal and meteorological variables such as survey month, daylight hours, or local temperature, as well as direct measures of light exposure, to examine how seasonal and environmental dynamics jointly moderate the relationship between outdoor activity and mental health.

## 5. Conclusions

This study set out to investigate whether embracing nature contributes to improved mental health among Chinese adults. Our analysis found that outdoor activity frequency significantly alleviates depressive mood and that intention exerts its protective influence entirely through increased behavioral engagement. The results directly answer our research question by confirming that the mental health benefits of embracing nature operate through actual engagement in outdoor activities rather than intention alone. This highlights the importance of interventions that not only encourage positive intentions but also facilitate the translation of these intentions into regular nature-based activities. Policymakers and practitioners should therefore focus on reducing barriers to outdoor engagement as part of strategies to promote mental health.

## Figures and Tables

**Table 1:** Variable definitions and measurements (N = 2583).

Variable	Measurement Method	Mean	SD	Min	Max
Dependent Variable					
Depression Mood	How often did you feel depressed in the past four weeks on a five-point scale (1: never, 5: always)	2.05	1.08	1	5
Independent Variable					
Outdoor Activity Intention	If possible, how much do you like engaging in outdoor activities in nature? 5-point scale (1: not at all, 5: very much)	3.26	1.23	1	5
Mediating Variable					
Outdoor Activity Frequency	In the past 12 months, how often did you participate in outdoor recreational activities? For example: hiking, bird watching, swimming, skiing, or other outdoor activities? 5-point scale (1: never, 5: almost every day)	2.35	1.27	1	5

**Table 2:** Variable descriptive statistics of respondents.

Characteristics	Category	N	Percentage (%)
Gender	Female	1401	54.2
	Male	1182	45.8
Age	18–29	361	14.0
	30–39	386	14.9
	40–49	374	14.5
	50–59	571	22.1
	60–69	452	17.5
	70–94	439	17.0
Marital status	Married	1780	68.9
	Other	803	31.1
Political status	Masses	2067	80
	Communist Party (Communist Youth League)/members of democratic parties	516	20
Family economic level	Far below average	228	8.8
	Below average	831	32.2
	Average	1290	49.9
	Above average	217	8.4
	Far above average	17	0.7
Physical health	Very unhealthy	131	5.1
	Somewhat unhealthy	324	12.5
	Average	679	26.3
	Somewhat healthy	932	36.1
	Very healthy	517	20
Nationality	Minority	194	7.5
	Han	2389	92.5
Education level	Primary school or below	842	32.6
	Junior high school or equivalent	718	27.8
	High school or equivalent	469	18.2
	Bachelor’s degree or above	554	21.4
Interview site	Neighborhood committee	1451	56.2
	Village committee	1132	43.8

**Table 3:** OLS Regression of Outdoor Activity Intention and Frequency on DM (N = 2583).

	Model 1 (DM)	Model 2 (DM)	Model 3 (Frequency)	Model 4 (DM)
Outdoor Activity Intention		−0.004 (0.016)	0.271 *** (0.020)	0.005 (0.016)
Outdoor Activity Frequency				−0.036 * (0.016)
Gender	−0.190 *** (0.038)	−0.190 *** (0.038)	0.101 * (0.047)	−0.187 *** (0.038)
Age	−0.006 *** (0.001)	−0.006 *** (0.001)	0.004 ** (0.002)	−0.005 *** (0.001)
Marital status	−0.108 * (0.042)	−0.108 * (0.042)	−0.048 (0.053)	−0.110 ** (0.042)
Political status	−0.017 (0.052)	−0.016 (0.052)	0.278 *** (0.066)	−0.006 (0.052)
Family economic status	−0.151 *** (0.025)	−0.150 *** (0.025)	0.155 *** (0.034)	−0.145 *** (0.025)
Physical condition	−0.400 *** (0.019)	−0.400 *** (0.019)	0.071 ** (0.026)	−0.397 *** (0.019)
Nationality	−0.032 (0.072)	−0.032 (0.072)	0.235 * (0.096)	−0.023 (0.072)
Education level	−0.037 (0.023)	−0.037 (0.024)	0.050 (0.031)	−0.035 (0.024)
Interview site	−0.164 *** (0.042)	−0.163 *** (0.042)	0.046 (0.053)	−0.162 *** (0.042)
Constant	4.484 *** (0.136)	4.494 *** (0.140)	0.382 * (0.183)	4.508 *** (0.140)
R^2^	0.219	0.219	0.125	0.221

* *p* < 0.05, ** *p* < 0.01, ***p < 0.001.

**Table 4:** Ordered Logistic Regression of Outdoor Activity Intention and Frequency on DM (N = 2583).

	Model 5	Model 6	Model 7
Outdoor Activity Intention		−0.027 (0.031)	−0.009 (0.032)
Outdoor Activity Frequency			−0.068 * (0.031)
Gender	−0.354 *** (0.076)	−0.355 *** (0.076)	−0.348 *** (0.076)
Age	−0.013 *** (0.003)	−0.013 *** (0.003)	−0.013 *** (0.003)
Marital status	−0.235 ** (0.083)	−0.233 ** (0.083)	−0.237 ** (0.083)
Political status	−0.079 (0.105)	−0.075 (0.105)	−0.056 (0.105)
Family economic status	−0.270 *** (0.050)	−0.270 *** (0.050)	−0.258 *** (0.050)
Physical condition	−0.791 *** (0.040)	−0.790 *** (0.040)	−0.786 *** (0.040)
Nationality	−0.091 (0.141)	−0.091 (0.141)	−0.080 (0.142)
Education level	−0.066 (0.046)	−0.061 (0.047)	−0.059 (0.047)
Interview site	−0.320 *** (0.082)	−0.315 *** (0.082)	−0.312 *** (0.083)
Cut 1	−5.371 *** (0.290)	−5.432 *** (0.298)	−5.471 *** (0.299)
Cut 2	−4.054 *** (0.282)	−4.115 *** (0.290)	−4.152 *** (0.291)
Cut 3	−2.403 *** (0.275)	−2.464 *** (0.284)	−2.498 *** (0.285)
Cut 4	−0.597 * (0.293)	−0.657 * (0.302)	−0.690 * (0.302)
Pseudo R^2^	0.206	0.206	0.207

* *p* < 0.05, ** *p* < 0.01, *** *p* < 0.001.

**Table 5:** Mediation Effect Analysis (N = 2583).

Path	Β	SE	LLCI	ULCI
Direct Effect	0.0053	0.0163	−0.0265	0.0372
Indirect Effect	−0.0096	0.0044	−0.0183	−0.0008
Total Effect	−0.0043	0.0157	−0.0351	0.0265

**Table 6:** Ordered Logistic Regression Results of the Effects of Outdoor Activity Intention and Interaction Frequency on DM (N = 1310).

	Model 8	Model 9	Model 10
Outdoor Activity Intention		−0.003 (0.044)	0.029 (0.045)
Outdoor Activity Frequency			−0.118 ** (0.044)
Gender	−0.485 *** (0.106)	−0.485 *** (0.106)	−0.479 *** (0.106)
Age	−0.010 * (0.004)	−0.010 * (0.004)	−0.009 * (0.004)
Marital status	−0.248 * (0.116)	−0.248 * (0.116)	−0.251 * (0.116)
Political status	−0.055 (0.148)	−0.054 (0.148)	−0.020 (0.149)
Family economic status	−0.209 ** (0.069)	−0.209 ** (0.069)	−0.197 ** (0.070)
Physical condition	−0.703 *** (0.056)	−0.703 *** (0.056)	−0.692 *** (0.056)
Nationality	0.065 (0.191)	0.065 (0.191)	0.075 (0.191)
Education level	−0.145 * (0.066)	−0.144 * (0.066)	−0.138 * (0.066)
Interview site	−0.216 (0.116)	−0.215 (0.117)	−0.220 (0.117)
Cut 1	−4.885 *** (0.406)	−4.893 *** (0.419)	−4.957 *** (0.420)
Cut 2	−3.556 *** (0.395)	−3.564 *** (0.409)	−3.622 *** (0.410)
Cut 3	−1.920 *** (0.388)	−1.928 *** (0.402)	−1.979 *** (0.403)
Cut 4	−0.263 (0.411)	−0.271 (0.424)	−0.318 (0.426)
Pseudo R^2^	0.188	0.188	0.193

* *p* < 0.05, ** *p* < 0.01, *** *p* < 0.001.

**Table 7:** Mediation Effect Analysis (N = 1310).

Path	Β	SE	LLCI	ULCI
Direct Effect	0.0180	0.0234	−0.0279	0.0640
Indirect Effect	−0.0171	0.0064	−0.0297	−0.0049
Total Effect	0.0009	0.0227	−0.0435	0.0454

## Data Availability

The data used in this study are from the 2021 wave of the Chinese General Social Survey (CGSS), which is publicly available at the CGSS data repository (http://cgss.ruc.edu.cn/). Researchers can access the dataset by registering on the official website.

## Abbreviations

The following abbreviations are used in this manuscript:

TPB	Theory of Planned Behavior
SRT	Stress Recovery Theory
ART	Attention Restoration Theory
DM	Depressive Mood
CGSS	Chinese General Social Survey
OLS	Ordinary Least Squares
